# Mechanisms Restricting Diffusion of Intracellular cAMP

**DOI:** 10.1038/srep19577

**Published:** 2016-01-22

**Authors:** Shailesh R. Agarwal, Colleen E. Clancy, Robert D. Harvey

**Affiliations:** 1 Department of Pharmacology University of Nevada School of Medicine Reno, NV 89557; 2 Department of Pharmacology University of California, Davis Davis, CA 95616.

## Abstract

Although numerous receptors stimulate cAMP production in a wide array of cells, many elicit distinct, highly localized responses, implying that the subcellular distribution of cAMP is not uniform. One often used explanation is that phosphodiesterases, which breakdown cAMP, act as functional barriers limiting diffusion. However, several studies refute the notion that this is sufficient, suggesting that phosphodiesterase-independent movement of cAMP must occur at rates slower than free diffusion. But, until now this has never been demonstrated. Using Raster Image Correlation Spectroscopy (RICS), we measured the diffusion coefficient of a fluorescently-labeled cAMP derivative (φ450-cAMP) as well as other fluorescent molecules in order to investigate the role that molecular size, cell morphology, and buffering by protein kinase A (PKA) play in restricting cAMP mobility in different cell types. Our results demonstrate that cytosolic movement of cAMP is indeed much slower than the rate of free diffusion and that interactions with PKA, especially type II PKA associated with mitochondria, play a significant role. These findings have important implications with respect to cAMP signaling in all cells.

The cAMP signaling pathway plays a fundamental role in regulating a variety of different activities in virtually every cell in our bodies. Cardiac myocytes are a classic example, where cAMP regulates multiple aspects of their electrical and mechanical activity. Membrane-associated adenylyl cyclases (ACs) generate the diffusible second messenger cAMP in response to activation of multiple G-protein coupled receptors, and subsequent activation of protein kinase A (PKA) by cAMP leads to phosphorylation of numerous downstream targets. However, different receptors can produce distinct responses even though they act through this common signaling pathway^1^,^2^,^3^,^4^. This behavior can be explained, in part, by the existence of signaling complexes that organize PKA and many of its relevant substrates in close proximity to one another^5^. However, this explanation alone is not sufficient. If cAMP were able to diffuse freely, any receptor would be able to activate PKA uniformly throughout the cell. To maintain the localized specificity of responses mediated by different receptors, it is necessary to restrict the movement of cAMP so that it only activates PKA close to its site of production. Even though cAMP signaling has long been the subject of investigation, the mechanisms that limit the movement of cAMP and contribute to compartmentalized behavior are not fully understood.

The common perception is that phosphodiesterases (PDEs), enzymes that break down cAMP, are enough by themselves to control the spread of cAMP by forming sinks or functional barriers to diffusion^6^,^7^,^8^,^9^,^10^. This impression is supported by experiments demonstrating that PDE inhibition can eliminate certain types of compartmentalized cAMP responses^11^,^12^. However, a number of modeling studies have concluded that while PDE activity is necessary for compartmentalized behavior, it alone is not sufficient to establish cAMP gradients^13^,^14^,^15^,^16^,^17^,^18^,^19^. These simulations suggest that for compartmentalized responses to occur, diffusion of cAMP in the cytoplasm must be much slower than the rate of free diffusion measured in dilute aqueous solutions. Only then is the amount of PDE activity found in cells sufficient to contribute to discrete locations where cAMP is metabolized faster than it can be replaced by diffusion from surrounding areas.

Determining how fast cAMP is moving inside the cell is essential to fully understanding the compartmentalized behavior of cAMP signaling. To this end, several groups have provided estimates for the cAMP diffusion coefficient in different cell types, including olfactory cilia, neurons, and neonatal cardiac myocytes^9^,^15^,^20^,^21^,^22^. The results suggest that the diffusion coefficient for cAMP in the cytoplasm of cells is between 136 and 780 μm^2^/s. However, these values are not significantly different from the rate of free diffusion of cAMP in aqueous solution, which has been measured at between 330 and 970 μm^2^/s^23^,^24^,^25^. In light of this discrepancy, computational models have predicted that certain types of compartmentalized behavior result from slow diffusion of cAMP near the plasma membrane due to physical barriers^15^,^26^, but what these physical barriers might be has not been determined. Mathematical modeling studies have also predicted that buffering due to binding of cAMP by the regulatory subunits of PKA may also limit diffusion within cells^15^,^26^. Thus, the idea that cAMP movement occurs at rates slower than free diffusion is still a theoretical prediction that has yet to be demonstrated experimentally.

One of the limitations of previous attempts to measure rates of cAMP diffusion in cells has been the reliance on indirect methods to monitor its movement. The main aim of the present study was to use newly developed membrane-permeable fluorescent cAMP analogues to directly monitor the movement of cAMP in intact cells using fluorescence correlation spectroscopy^27^,^28^. Specifically, we used raster image correlation spectroscopy (RICS) to estimate the diffusion coefficient of 8-[Pharos-450]-cAMP (φ450-cAMP ) a fluorescently-labeled cAMP analogue^29^, as well as other fluorescent molecules, in both cardiac myocytes and HEK 293 cells, to investigate the role that molecule size, cell morphology, and PKA buffering play in restricting cAMP diffusion.

## Results

**Figure 1:**
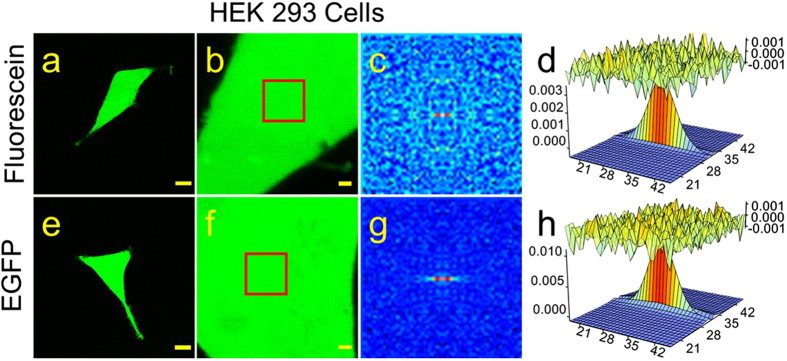
RICS analysis of fluorescein and EGFP in HEK293 cells. Representative confocal images of fluorescein (**a,b**) and EGFP (**e,f**) in HEK293 cells at low (**a,e**, scale bar 10 μm) and high (**b,f**, scale bar 1 μm) magnification. (**c,g**) Average of the spatial correlation calculated for a region of 64 × 64 pixels (indicated by the red box in **b,f**) for each of 100 images of fluorescein (**c**) and EGFP (**g**) in HEK293 cells. Fit of the correlation functions for fluorescein (**d**) and EGFP (**h**). The plot at the top of those panels represents the difference between the autocorrelation and the fit.

Raster Image Correlation Spectroscopy (RICS) analysis of confocal images was used to measure the mobility of fluorescent molecules in intact cells. Initial experiments were conducted in HEK 293 cells loaded with fluorescein (MW 332) using the membrane permeable diacetate derivative (Fig. 1a,b). Diffusion coefficients were obtained from autocorrelation functions as described in Methods and Materials (Fig. 1c,d). From this analysis we obtained a value of 76 ± 3.9 μm^2^/sec (n = 4). This is approximately four fold slower than the rate of free diffusion of fluorescein in aqueous solution (300 μm^2^/s)^30^, but it is consistent with previously published values measured in intact cells^31^. We then used the same method to calculate the diffusion coefficient of EGFP (32.7 kDa) expressed in HEK293 cells (Fig. 1e–h). The value we obtained, 31 ± 1.8 μm^2^/sec (n = 8) (Fig. 1g,h), again was significantly slower than the value for free diffusion of the same molecule in aqueous solution (see Supplementary Fig. S1). It was also significantly slower than the diffusion coefficient for fluorescein in these cells, which is consistent with previous estimates^29^. These results support the idea that just as in an aqueous solution, the size of the molecule affects the rate of free diffusion in the cytosol of intact cells.

**Figure 2:**
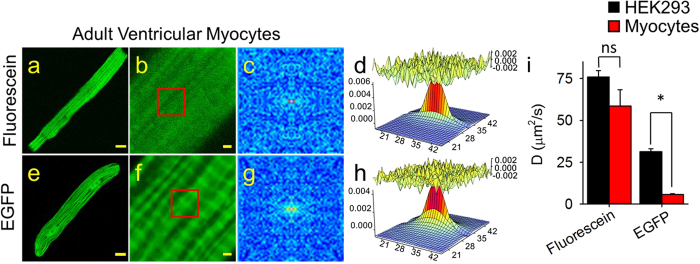
RICS analysis of fluorescein and EGFP in adult ventricular cardiac myocytes. Representative confocal images of fluorescein (**a,b**) and EGFP (**e,f**) in myocytes at low (**a,e**, scale bar 10 μm) and high (**b,f**, scale bar 1 μm) magnification. (**c,g**) Average of the spatial correlation calculated for a region of 64 × 64 pixels (indicated by the red box in **b,f**) for each of 100 images of fluorescein (**c**) and EGFP (**g**) in myocytes. Fit of the correlation functions for fluorescein (**d**) and EGFP (**h**). The plot at the top of those panels represents difference between the autocorrelation and the fit. (**i**) Bar plots (average ± s.e.m.) of the diffusion coefficient (*D*) values obtained for fluorescein and EGFP in HEK293 cells (black bars) and cardiac myocytes (red bars). *p < 0.05, ns = not significant. HEK293 cells: n = 4–8, myocytes: n = 7–22.

We next addressed the question of whether or not the rate of cytosolic diffusion of these same molecules might be affected by the more complex morphology of an adult ventricular myocyte. When cardiac myocytes were loaded with fluorescein, the fluorophore was distributed throughout the cell (Fig. 2a,b). Furthermore, RICS analysis (Fig. 2c,d) revealed a diffusion coefficient (59 ± 9.7 μm^2^/sec, n = 7) similar to that found in HEK cells (Fig. 2i). In contrast, expression of EGFP in cardiac myocytes resulted in a distinctly non-uniform pattern of fluorescence (Fig. 2e,f). This suggests that the movement of EGFP may be more restricted than that of fluorescein. Consistent with this idea, RICS analysis (Fig. 2g,h) revealed that EGFP has a diffusion coefficient of 5.8 ± 0.38 μm^2^/sec (n = 22) in cardiac myocytes. This is not only significantly slower than the diffusion coefficient of fluorescein in cardiac myocytes, it is also significantly slower than the diffusion coefficient of EGFP in HEK cells (Fig. 2i). These data confirm the idea that not only do larger molecules diffuse more slowly than smaller molecules in any given cell type^32^, but that the movement of large molecules, like EGFP, may also be affected by the morphology of cell.

**Figure 3:**
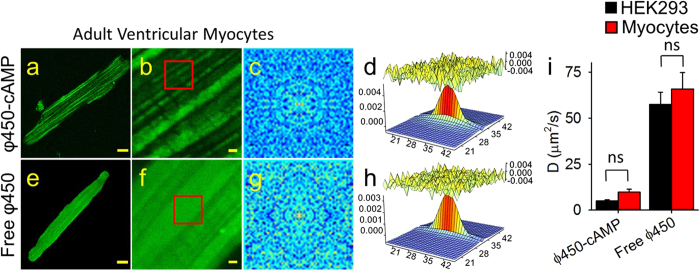
RICS analysis of φ450-cAMP and free φ450 in adult ventricular cardiac myocytes. Representative confocal images of φ450-cAMP (**a,b**) and free φ450 (**e,f**) in myocytes at low (**a,e**, scale bar 10 μm) and high (**b,f**, scale bar 1 μm) magnification. (**c,g**) Average of the spatial correlation calculated for a region of 64 × 64 pixels (indicated by the red box in **b,f**) for each of 100 images of φ450-cAMP (**c**) and free φ450 (**g**) in myocytes. (**d,h**) Fit of the correlation functions. The plot at the top of those panels represents the difference between the autocorrelation and the fit. (**i**) Bar plots (average ± s.e.m.) of the diffusion coefficient (D) values obtained for φ450-cAMP and free φ450 in HEK293 cells (black bars) and cardiac myocytes (red bars). *p < 0.05, ns = not significant. HEK293 cells: n = 8–12, myocytes: n = 9–37.

Next we attempted to estimate the diffusion coefficient of cAMP in intact cells. Previous studies have demonstrated that cAMP signaling is compartmentalized in both HEK cells^16^,^18^,^26^,^33^ and adult ventricular myocytes^2^,^3^,^4^,^8^,^12^. Furthermore, computational analysis suggests that this behavior can only be explained if movement of cAMP in the cytosol occurs at rates that are significantly slower than free diffusion (~300 μm^2^/sec)^13^,^14^,^15^,^16^,^18^,^19^,^26^. Consistent with this prediction, the diffusion coefficient of fluorescein, which is nearly identical in size to cAMP (MW 326), is much slower in both cell types than it is in aqueous solution. To estimate the diffusion coefficient of cAMP, we used a version of this molecule labeled with a fluorescent pharos tag. The high lipophilicity of pharos dyes renders the corresponding cAMP analogues membrane permeable^27^. Loading ventricular myocytes with cAMP labeled with a green pharos tag (φ450-cAMP) resulted in a striated pattern of fluorescence running longitudinally or parallel to the long axis of the cell (Fig. 3a,b). This is distinctly different from the fluorescence pattern observed with either fluorescein or EGFP (see Fig. 2). RICS analysis (Fig. 3c,d) revealed that the diffusion coefficient of φ450-cAMP (9.7 ± 1.6 μm^2^/sec) (Fig. 3i) was similar to that of EGFP (see Fig. 2). However it is unlikely that this can be explained by its size, since the molecular weight of φ450-cAMP (MW ≈ 720) is only ~2% of that of EGFP. In sharp contrast, incubation of cardiac myocytes with the free dye (φ450) resulted in a more uniform pattern of fluorescence (Fig. 3e,f), similar to that observed with fluorescein (see Fig. 2). The free dye (MW ≈ 390) is similar in size to fluorescein. Consistent with that, RICS analysis (Fig. 3g,h) revealed a diffusion coefficient of 66 ± 1.7 μm^2^/sec (n = 14) (Fig. 3i), which is similar to that of fluorescein (see Fig. 2i). These results indicate that the fluorescence pattern observed with φ450-cAMP is due to a binding interaction involving cAMP and not due to the attached dye.

The smaller size of φ450-cAMP and its distribution pattern, which is distinctly different from that of EGFP, suggests that its slow diffusion coefficient is not simply due to the more complex architecture of cardiac myocytes. To examine this question, we measured the diffusion coefficients of φ450-cAMP in the morphologically simpler HEK 293 cells. When loaded with φ450-cAMP, these cells exhibited a non-uniform, punctate pattern of fluorescence (Supplementary Fig. S3a,b), and RICS analysis (Supplementary Fig. S3c,d) revealed a diffusion coefficient of 5.0 ± 0.60 μm^2^/sec (n = 12), which is similar to that of φ450-cAMP in cardiac myocytes (Fig. 3i). When loaded with free dye, HEK 293 cells exhibited a uniform fluorescence pattern (Supplementary Fig. S3e,f), similar to that of fluorescein (see Fig. 1). Like fluorescein, RICS analysis (Supplementary Fig. S3g,h) revealed a diffusion coefficient of 57 ± 6.6 μm^2^/sec (n = 8) (Fig. 3i). These findings are consistent with the distribution pattern observed in these cells when using red pharos dye with (φ575-cAMP) and without cAMP (φ575) attached^27^. They also support the idea that the slow rate of diffusion of φ450-cAMP derivative is not a function of cell morphology.

**Figure 4:**
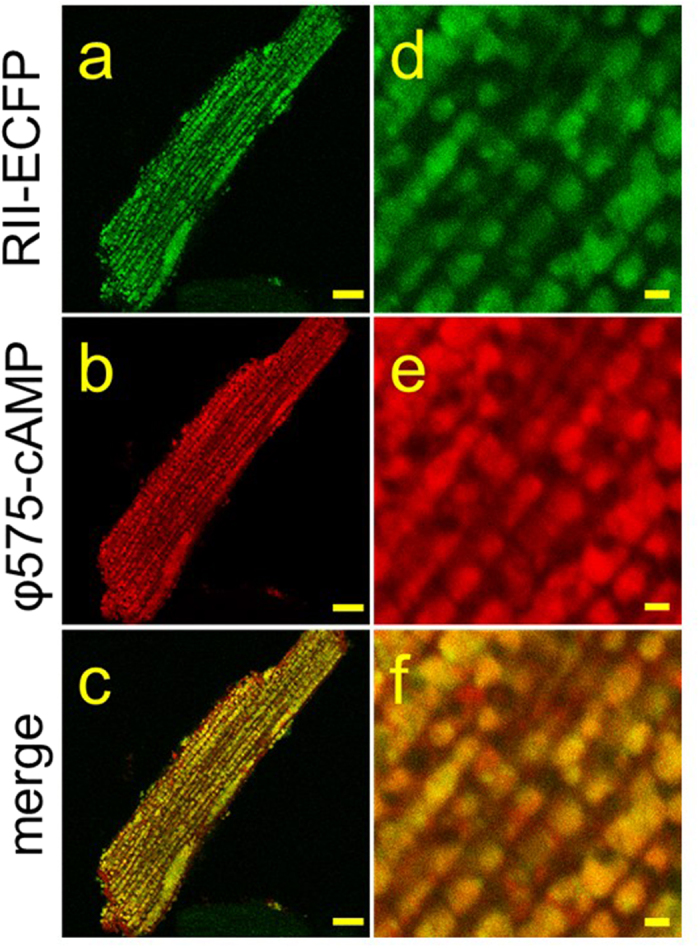
Co-localization of φ575-cAMP with the type II regulatory subunit (RII) of PKA in adult ventricular cardiac myocytes. Confocal images of myocytes expressing RII-ECFP (**a,d**) or loaded with φ575-cAMP (**b,e**) at low (left panels, scale bar 10 μm) and high (right panels, scale bar 1 μm) magnification. The merged images (**c,f**) demonstrate a high degree of co-localization between RII-ECFP and φ575-cAMP. This experiment represents an example of cells in which RII-ECFP exhibited a longitudinal striated pattern. PCC 0.41 ± 0.014, tM1 0.55 ± 0.014, tM2 0.71 ± 0.014, n = 9.

Previous studies have suggested that buffering by protein kinase A (PKA) may be a mechanism controlling the spread of cAMP within cells^15^,^19^,^34^,^35^. If the diffusion of φ450-cAMP is indeed controlled by binding to the regulatory subunit of PKA, then we should see co-localization of the two. The extremely slow rate of diffusion we measured suggests that cAMP may be interacting with regulatory subunits that are anchored to fixed structures, and A kinase anchoring proteins (AKAPs) are known to create signaling complexes by anchoring PKA together with target proteins in cardiac myocytes^5^. While there are AKAPs that bind type I as well as type II PKA, type II is the predominant subtype found in the particulate fraction of cardiac myocytes^36^. Therefore, we looked for co-localization of cAMP and the type II regulatory subunit tagged with ECFP (RII-ECFP) (Fig. 4). For these experiments we used cAMP labeled with a red pharos dye (φ575-cAMP). Most cardiac myocytes expressing the RII-ECFP construct exhibited a longitudinal striated pattern (Fig. 4a,d), similar to that observed in cells loaded with either φ450-cAMP (see Fig. 3) or φ575-cAMP (Fig. 4b,e). Although co-localization does not prove direct physical interaction, calculation of the Pearson’s (PCC) and Manders Correlation Coefficients (MCC) indicate that there is a high degree of correlation between the location of the φ575-cAMP and RII-ECFP in our merged images (Fig. 4c,f). In a smaller fraction of myocytes, RII-ECFP also exhibited a horizontal pattern of expression that also co-localized with φ575-cAMP (Supplementary Fig. S4).

**Figure 5:**
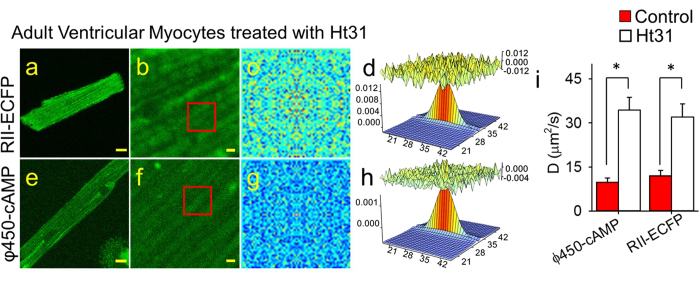
RICS analysis of φ450-cAMP and RII-ECFP in adult ventricular cardiac myocytes treated with St-Ht31 peptide. Representative confocal images of St-Ht31-treated myocytes expressing RII-ECFP (**a,b**) or loaded with φ450-cAMP (**e,f**) at low (**a,e**, scale bar 10 μm) and high (**b,f**, scale bar 1 μm) magnification. (**c,g**) Average of the spatial correlation calculated for a region of 64 × 64 pixels (indicated by the red box in **b,f**) for each of 100 images of RII-ECFP (**c**) and φ450-cAMP (**g**) in St-Ht31-treated myocytes. Fit of the correlation functions for RII-ECFP (**d**) and φ450-cAMP (**h**). The plot at the top of those panels represents the difference between the autocorrelation and the fit. (**i**) Bar plots (average ± s.e.m.) of the diffusion coefficient (*D*) values obtained for RII-ECFP and φ450-cAMP in control (red bars) or St-Ht31-treated (open bars) myocytes (*p < 0.05. RII-ECFP expressing cells: n = 8–11, φ450-cAMP loaded cells: n = 15–37).

In addition to demonstrating that there is co-localization of RII-ECFP and φ575-cAMP, we also performed RICS analysis on cells expressing RII-ECFP alone (Supplementary Fig. S5). The results indicate that the diffusion coefficient of the RII construct is 12 ± 1.9 μm^2^/sec (n = 11), which is similar to the diffusion coefficient of φ450-cAMP (see Fig. 5i). These results are consistent with the hypothesis that the mobility of cAMP is regulated by its interaction with the regulatory subunit of type II PKA.

If indeed the mobility of φ450-cAMP is limited by binding to RII subunits of PKA, then disrupting the interaction between RII and AKAP should affect the mobility of both RII-ECFP as well as φ450-cAMP. To test this idea, we exposed cardiac myocytes to a membrane-permeable form of the Ht31 peptide (St-Ht31), which mimics the region of the AKAP protein known to bind RII subunits and prevents PKA anchoring^3^,^11^. When cardiac myocytes expressing RII-ECFP (Fig. 5a,b) were incubated with St-Ht31, the fluorescence pattern was more diffuse than that observed in non-treated cells (see Fig. 4a, d). Correspondingly, RICS analysis of RII-ECFP (Fig. 5c,d) revealed that the diffusion coefficient increased by about 3 fold to 32 ± 4.5 μm^2^/sec (n = 15) (Fig. 5i). Similarly, in myocytes loaded with φ450-cAMP (Fig. 5e,f), addition of St-Ht31 resulted in a fluorescence pattern more diffuse than that seen in non-treated cells (see Fig. 3a, b), and RICS analysis (Fig. 5g,h) revealed that the diffusion coefficient increased significantly to 34 ± 4.4 μm^2^/sec (n = 8) (Fig. 5i). In cells exposed to the control peptide, St-Ht31P, which does not inhibit the interaction between the RII subunit and AKAP, the diffusion coefficient of φ450-cAMP was 10 ± 1.1 μm^2^/sec (n = 16), which is not significantly different from untreated cells.

To further test the idea that the pharos-tagged cAMP analogs used in this study are interacting with PKA, we also conducted confocal imaging experiments in cardiac myocytes loaded with 8-φ575-2′-O-Me-cAMP, which is a cAMP analog that does not bind the regulatory subunits of PKA^37^. Unlike the pattern observed in cells loaded with φ450-cAMP, cells loaded with 8-φ575-2′-O-Me-cAMP exhibited a more diffuse pattern of fluorescence (Supplementary Fig. S6). These data provide additional support for the idea that φ450-cAMP and φ575-cAMP are binding to PKA and that this buffering effect plays a significant role in limiting the diffusion of cAMP in cardiac myocytes.

It has been reported that pharos-labeled cAMP compounds are resistant to hydrolysis by PDE type 4^27^. However, cardiac myocytes also express PDE2 and PDE3 activity^8^. Therefore, it is possible that PDE hydrolysis involving one of these other isoforms may have affected the movement of φ450-cAMP in these cells. However, in myocytes exposed to 100 μM IBMX, a non-selective PDE inhibitor, the diffusion coefficient of φ450-cAMP was 12 ± 2.2 μm^2^/s (n = 14), which is not significantly different from what was observed in untreated cells. This supports the notion that PDE2, 3, and 4 activity is not responsible for slowing the movement of φ450-cAMP. It also suggests that any increase in endogenous cAMP levels produced by IBMX was not enough to displace a detectable fraction of the bound φ450-cAMP. These results do not, however, rule out the possibility that PDE activity can affect the rate of diffusion of endogenous cAMP.

**Figure 6:**
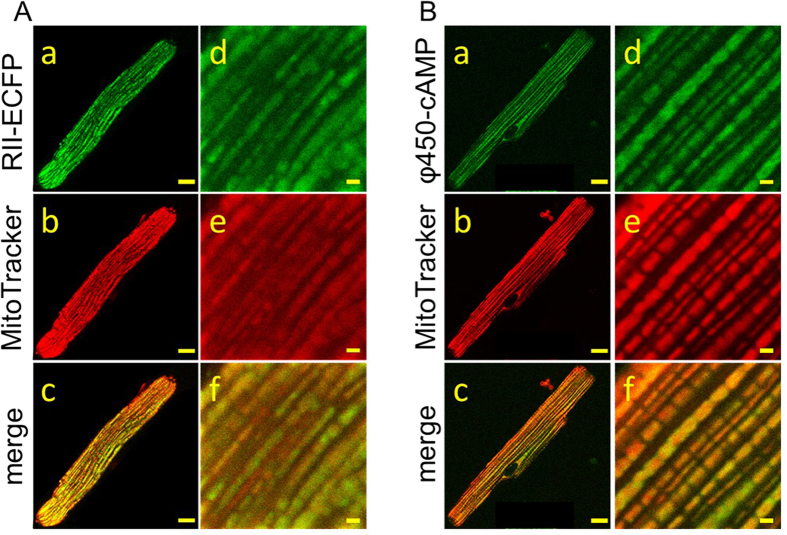
Co-localization of the type II regulatory subunit (RII) of PKA or φ450-cAMP with MitoTracker red in cardiac myocytes. Representative confocal images of myocytes expressing RII-ECFP (A) or loaded with φ450-cAMP (**B**) at low (**a–c**, scale bar 10 μm) and high (**d–f**, scale bar 1 μm) magnification. Mitochondria labeled with MitoTracker red show a longitudinal striated pattern (**b,e**). The merged images demonstrate a high degree of co-localization of RII-ECFP (Ac, Af) or φ450-cAMP (Bc, Bf) with MitoTracker red. PCC 0.28 ± 0.017, tM1 0.49 ± 0.018, tM2 0.60 ± 0.017, n = 7 (panel A). PCC 0.22 ± 0.0049, tM1 0.39 ± 0.0064, tM2 0.65 ± 0.0066, n = 13 (panel B).

Ultrastructure studies using electron microscopy and morphometry have revealed that mitochondria are arranged in a longitudinal pattern between the myofibrils of cardiac ventricular myocytes^38^, where they occupy ~30% of the intracellular volume^39^,^40^. Furthermore, cardiac myocytes express AKAPs that target the binding of PKA to mitochondria^5^. This suggests that the longitudinal pattern exhibited by φ450-cAMP could be due to its interaction with PKA targeted to mitochondria. To test this hypothesis, we looked for co-localization of both RII-ECFP and φ450-cAMP with mitochondria labeled with MitoTracker Red. As shown in Fig. 6, MitoTracker exhibits a longitudinal striated pattern, similar to the pattern exhibited in cells expressing RII-ECFP (Fig. 6A) or cells loaded with φ450-cAMP (Fig. 6B). Furthermore, quantitative correlation analysis of merged images of MitoTracker red with either RII-ECFP (Fig. 6A) or φ450-cAMP (6B) demonstrated a high degree of co-localization. These results support the hypothesis that PKA associated with mitochondria play an important role in buffering cAMP in cardiac myocytes. We also found that φ450-cAMP exhibited a high degree of co-localization with mitochondria in HEK 293 cells (Supplementary Fig. S7).

## Discussion

Intracellular diffusion is affected by three independent factors: the fluid-phase viscosity of the cytoplasm, collisional interactions caused by molecular crowding, and binding interactions with mobile and fixed molecules in cells^32^. Fluid-phase viscosity is defined as the viscosity sensed by a solute in the absence of any interaction with other molecules or structures. Various studies have demonstrated that the cytoplasmic fluid-phase of mammalian cells is only slightly more viscous than water and that this difference does not play a major role in affecting the translational movement of molecules^32^,^41^. Nevertheless, the diffusion coefficient of small molecules measured in the cytoplasm of cells is typically 3- to 8-fold slower than it is in aqueous solution^32^,^42^. That is consistent with the 4 to 5 fold slower rate of diffusion we found for fluorescein in HEK 293 cells and adult ventricular myocytes in the present study. Such slowing of diffusion has been attributed to collision with other solutes, macromolecules, and cellular structures as a consequence of macromolecular crowding^32^.

We also found that the diffusion coefficient of EGFP was 3 fold slower in the cytoplasm of HEK 293 cells than it was in aqueous solution. Again, this is consistent with previous reports and can be attributed to slowing due primarily to molecular crowding^32^,^43^,^44^. On the other hand, we found that the diffusion coefficient of EGFP was reduced approximately 15-fold in cardiac myocytes, where there is a highly ordered and complex arrangement of contractile elements, transverse-tubules, and intracellular organelles like mitochondria and the sarcoplasmic reticulum^39^,^45^. While the structural organization of cardiac myocytes does not seem to affect the diffusion of small molecules like fluorescein, it may hinder movement of larger molecules like EGFP via a mechanism referred to as solute “screening”^46^. It is interesting to note that although both EGFP and φ450-cAMP exhibited a longitudinal striated pattern in cardiac myocytes, φ450-cAMP appeared to be associated primarily with mitochondria (see Fig. 6b) while EGFP was actually excluded from the mitochondrial space (Supplementary Fig. S8).

Based on size considerations alone, one would predict that cytoplasmic rate of cAMP diffusion should be similar to fluorescein, or 4- to 5-fold slower than the rate of free diffusion. However, previous attempts to measure the intracellular diffusion coefficient of cAMP have suggested that it actually moves at rates equal to or faster than free diffusion^9^,^15^,^20^,^21^,^22^. The reason may have to do with the approach used to come up with those estimates. They all involved indirect methods utilizing the response of endogenous cAMP ligands, such as nucleotide gated (CNG) ion channels or expressed cAMP biosensors. Chen *et al.*^21^ estimated the diffusion coefficient in olfactory cells from the time course of CNG channel current activation by extracellular cAMP as it diffused into cilia following their excision using a patch pipette. It is conceivable in this case that the dialysis of the cilia upon excision not only allowed cAMP to enter, but factors potentially slowing diffusion to be lost. More recently, the development of genetically encoded biosensors has made it possible to study the movement of cAMP in intact cells. In these studies, changes in the biosensor response were monitored at various locations in a cell following the release or production of cAMP at a distal site. Again, the movement of cAMP was not being measured directly, and while in some cases the biosensor response was a function of cAMP binding, in others it was actually responding to phosphorylation by PKA^9^,^15^,^20^,^22^.

In the present study, our estimates of the cAMP diffusion coefficient were based on direct measurement of cAMP movement using φ450-cAMP. Although the size of this molecule (MW ≈ 720) is slightly more than double that of cAMP itself, calculations using the Stokes-Einstein equation predict a diffusion rate of 50 μm^2^/sec, which is only slightly slower than the diffusion coefficients we obtained for fluorescein or the free-pharos dye, both of which are the same size as cAMP alone. Using the RICS technique, we determined the diffusion coefficient for φ450-cAMP was 9.7 ± 1.6 μm^2^/sec and 5.0 ± 0.60 μm^2^/sec in myocytes and HEK293 cells, respectively. These values are significantly slower than the predicted rate of free diffusion. They are also consistent with those predicted to be necessary to produce cAMP gradients within cells^35^.

Previous studies have suggested that diffusion of cAMP may be affected by PKA buffering^15^,^26^,^34^,^35^. We found that φ450-cAMP co-localized with the RII subunit of PKA. Furthermore, disrupting RII interactions with AKAP altered not only the distribution pattern and mobility of the RII subunit, it had an identical effect on the distribution pattern and mobility of φ450-cAMP. These results support the idea that buffering by the RII subunit of PKA plays a significant role in determining the rate of cAMP diffusion in cardiac myocytes. This is consistent with the findings of Nikolaev *et al.*, who demonstrated that disrupting RII-AKAP interactions also interrupts β_2_-adrenergic receptor-mediated localized cAMP responses^34^.

When using fluorescence fluctuation spectroscopy techniques the fluorophore concentration needs to be kept low (between approximately 0.1 and 1 μM) to accurately determine its diffusion coefficient^47^,^48^. The intracellular concentrations of fluorophores used in our experiments are listed in Supplementary Table S1. In cardiac myocytes, the concentration of φ450-cAMP varied between 180 and 960 nM. These levels are consistent with those thought to exist in type II PKA signaling domains following agonist stimulation, however, they are closer to estimated basal levels of endogenous cAMP throughout much of the remainder of the cell^49^.

It has been estimated that mammalian cardiac myocytes contain 0.5 to 1 μM PKA, which corresponds to a cAMP buffering capacity of up to 2 μM^19^,^50^. Under these conditions, where the number of binding sites exceeds the ligand concentration, most of the φ450-cAMP should be bound^51^. Consistent with this, it has been reported that a large portion of cellular cAMP is bound to the RII subunit of PKA under basal conditions^36^. Still, a certain fraction of the φ450-cAMP in our experiments is likely to have been located in microdomains where its diffusion is not affected by interactions with PKA. However, even in locations such as those near the plasma membrane, the mobility of cAMP has been reported to be restricted^15^,^52^. Nevertheless, RICS calculates diffusion coefficients over a relatively large area (3.2 μm^2^). Therefore, our estimate of φ450-cAMP mobility is certain to reflect diffusion coefficients across different microdomains. In addition, at least one previous study has suggested that the lipophilicity of cAMP analogues may increase the apparent rate of diffusion^26^, which means that we may have actually underestimated the potential influence of buffering on cAMP diffusion.

Another interesting finding of the present study is that type II PKA associated with mitochondria appears to play a particularly important role in buffering cAMP in cardiac myocytes. This is supported by the results of the co-localization experiments using either φ450-cAMP or RII-ECFP and Mito-Tracker Red (see Fig. 6). The same also appears to be true in HEK 293 cells. Not only was the diffusion coefficient of φ450-cAMP similar to that measured in cardiac myocytes, we found that φ450-cAMP co-localizes with MitoTracker Red in these cells as well (Fig. S7). Furthermore, it has been shown that PKA is enriched in the outer mitochondrial membrane of HEK293 cells^53^,, where φ575-cAMP co-localizes with the RII subunit of PKA^27^.

At least three different AKAPs (D-AKAP1, D-AKAP2 and Rab32) are associated with mitochondria^5^,^54^. D-AKAPs can actually anchor both type I and type II PKA. Although our results clearly demonstrate a role for the regulatory subunit of type II PKA in buffering the movement of cAMP, we cannot rule out the possibility that type I subunits are also involved. In either case, it is not clear if these regulatory subunits are associated with catalytic subunits, in which case cAMP binding would not necessarily be associated with PKA activation. However, Lefkimmiatis *et al.*^53^ have reported that PKA activity at the outer mitochondrial membrane of some cells is persistently active. In this case, cAMP binding could also play a role in regulating mitochondrial function^54^.

In cardiac myocytes, D-AKAP2 mutations affecting PKA binding affinity are associated with life threatening cardiac arrhythmias and sudden cardiac death^55^. Although alterations in mitochondrial function could be involved, sympathetic and parasympathetic stimulation control the electrical activity of the heart through the tightly orchestrated regulation of a number of different ion channels and transporters by mechanisms that involve cAMP signaling. It is conceivable that changes in PKA anchoring to mitochondria could alter this delicate balance by affecting compartmentation of cAMP signaling. Further studies will be needed to elucidate the role of mitochondrial sequestration of cAMP in all cells, not just cardiac myocytes.

While it appears that a majority of φ450-cAMP and/or RII-ECFP was associated with mitochondria, the Pearson’s and Mander’s Correlation Coeffcients indicates that there is also a significant fraction that does not colocalize with Mitotracker Red (Fig. 6, and S7). This suggests that cAMP buffering by type II PKA occurs at sites other than just those associated with mitochondria.

## Methods and Materials

### Cell isolation and culture

Ventricular myocytes were isolated from the hearts of adult male Sprague Dawley rats (250–300 g) as described previously^2^. The protocol used was in accordance with the Guide for the Care and Use of Laboratory Animals as adopted by National Institutes of Health and approved by the Institutional Animal Care and Use Committee at the University of Nevada, Reno.

Myocytes used for imaging experiments on the day of isolation were resuspended and plated in extracellular solution (ES) containing (in mM) 137 NaCl, 5.4 KCl, 0.5 MgCl_2_, 1.0 CaCl_2_, 0.33 NaH_2_PO_4_, 5 HEPES, 5.5 glucose, pH 7.4 and maintained at 37 °C. For imaging experiments that required transduction with adenovirus to express the type II regulatory subunit of PKA (Ad-RII-ECFP) or enhanced green fluorescent protein (Ad-EGFP), myocytes were resuspended in minimum essential medium (MEM) containing insulin-trasferrin-selenium (1X), bovine serum albumin (1 mg/ml), 2,3-butanedione monoxime (5 mM), penicillin (100 units/ml), and streptomycin (100 μg/ml). After incubation for 2 h at 37 °C, the cells were transduced with adenovirus constructs using a multiplicity of infection (MOI) of 50-100. Before imaging, myocytes were transferred to 35 mm glass-bottom FluoroDishes (World Precision Instruments, Inc.).

Human embryonic kidney (HEK) 293 cells were grown in FluoroDishes using DMEM containing 10% fetal bovine serum, penicillin, and streptomycin. For experiments involving EGFP, cells were transduced using Ad-EGFP at an MOI of 1–10.

### Confocal Imaging and RICS analysis

Experiments were conducted using 8-[Pharos-450]-cAMP (φ450-cAMP), 8-[Pharos-575]-cAMP (φ575-cAMP) and 8-[Pharos-575]-2′-O-Me-cAMP (φ575-O-Me-cAMP). These consist of cAMP or cAMP derivative labeled with a small (MW ≈390) pharos fluorophore. The high lipophilicity of these fluorophores renders the cAMP analogue membrane permeable. Previous studies have demonstrated that attachment of the fluorophore does not affect its ability to activate PKA. However, it has been reported to prevent PDE hydrolysis^27^.

Myocytes or HEK293 cells were loaded with membrane permeable fluorophores by incubating in ES containing φ450-cAMP or φ575-cAMP (200 μM for 1 hour), φ450 free dye (200 μM for 10 minutes), φ575-O-Me-cAMP (70 μM for 1 hour), fluorescein diacetate (100 nM for 10 minutes) or MitoTracker Red (1 μM for 10 minutes) at 37 °C before use. For experiments involving the use of membrane-permeable InCELLect AKAP St-Ht31 and St-Ht31P control peptide^3^,^56^, cells were incubated in ES containing the peptide (20 μM for 1 hour) at 37 °C. Cells expressing RII-ECFP and EGFP were imaged 48-72 hours post adenoviral transduction. Before imaging, cells were washed twice with ES.

Raster Image Correlation Spectroscopy (RICS) was used to measure the diffusion coefficient (*D*) of various fluorophores using the Globals for Images SimsFCS software package developed by the Laboratory of Fluorescence Dynamics at the University of California, Irvine^29^. Briefly, the fluorescence emitted from the focal volume excited by the laser beam of a scanning confocal microscope is measured on a pixel by pixel basis. Fluctuations in the measured intensity provide information about the mobility of the fluorophore. The diffusion coefficient can be obtained from a fit of the autocorrelation function (*G*) based on the pixel size, pixel dwell time, line scan time, and the focal volume^29^.

Confocal images were obtained using an Olympus Fluoview 1000 microscope in photon count mode with an oil immersion objective (60X, 1.42 NA). The confocal aperture was fixed at 1 Airy unit (100 μm)^57^. For RICS analysis, 100 images of 256 × 256 pixels (12.8 μm X 12.8 μm) were captured at 16.4X zoom, corresponding to a pixel size of 50 nm, using a laser power of <1%. A pixel dwell time of 10 μs and line scan time of 3.68 ms were used. Images were obtained using the 488 nm line of argon laser and a BA505-525 emission filter.

Focal volume was calculated from the focal volume waist (ω_0_) or the radius of the laser beam point spread function (PSF), which is a function of the optics of the microscope. It can also be affected by the alignment of the laser. Therefore, to control for possible day-to-day variations in laser alignment, ω_0_ was calibrated at the beginning of each day’s experiments by performing RICS analysis of a aqueous solution containing fluorescein (50 nM) in Tris buffer (100 mM, pH 9) (Supplementary Fig. S1a–c). Background fluorescence was removed by subtracting the average fluorescence of the entire sequence from each frame. Autocorrelation functions were calculated for each of the 100 images and then averaged and fitted using the known diffusion coefficient for fluorescein (300 μm^2^/sec)^30^ in order to determine ω_0_. Typical values were in the range of 0.25–0.35 μm. The axial length (ω_z_) of the PSF was assumed to be 3 times ω_0_^28^,^30^,^57^,^58^

To verify the accuracy of the ω_0_ values obtained, we performed RICS analysis on another fluorophore, EGFP, for which the diffusion coefficient is also known (Supplementary Fig. S1d-f). Stock solution containing EGFP was prepared in Tris buffer and aliquots were stored at -80 °C. To avoid spontaneous adsorption of EGFP, the cover glass was pre-coated with 1% bovine serum albumin (BSA) for 1 hour at 37 °C^30^. Stock solutions were diluted in Tris buffer containing 1% BSA. The values of ω_0_ obtained from RICS analysis of fluorescein were then used to fit the autocorrelation of the images of EGFP in Tris buffer. We obtained a value of 87 ± 5.3 μm^2^/sec (n = 9) (Supplementary Fig. S1g), which is consistent with what has been previously reported^28^,^29^,^43^.

For RICS analysis of fluorophores inside cells, spatial correlation was performed for a selected region (64 × 64 pixels) of the image. Immobile or background fluorescent features were removed by subtracting a running average of 10 frames from each image^29^. Use of the cAMP analogue φ450-cAMP, which is excitable at the same wavelength as fluorescein and EGFP, allowed us to use calibration measurements made with those fluorophores.

The molar concentration (C) of fluorophore was calculated using equation (1)^47^,^48^,^59^:


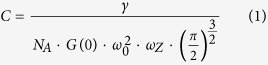


where *γ* is a geometric factor that depends on the shape of the excitation volume, N_A_ is Avogadro’s number, G(0) is the value of autocorrelation function at time zero obtained from RICS analysis, ω_0_ is the radial waist and ω_z_ is the axial waist of the PSF. Assuming a 3D Gaussian PSF profile, the value of *γ* is 0.3536. The intracellular concentration of the different fluorophores used in our experiments is listed in Supplementary Table S1. The diffusion coefficients did not change significantly over the range of concentrations used.

Fluorescence imaging and co-localization experiments involving MitoTracker Red and φ575-cAMP were performed using the 543 nm line of a HeNe laser and a BA560-660 emission filter. Images of 1024 × 1024 pixels at 2X (low magnification) and 16.4X (high magnification) were captured sequentially to prevent any bleed through in the signals. Each experiment was performed on at least three different days.

The degree of fluorophore co-localization was quantified by calculating the Pearson’s correlation coefficient (PCC) (eq. (2)):


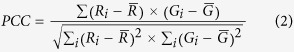


where R_i_ and G_i_ refer to the intensity values of the red and green channels, respectively, of pixel i, and 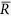 and 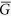 refer to the mean intensities of the red and green channels, respectively, within the region of interest. PCC values range from 1 for perfect co-localization, to -1 for perfect inverse co-localization^60^:

The degree of fractional overlap of fluorophores was quantified by calculating the Mander’s Co-localization Coefficients (MCC) (eqs. (3) and (4)):


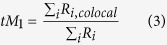


where tM_1_ = fraction of red-emitting pixels that also emit green, R_i,colocal_ = R_i_ if G_i_ > 0 and R_i,colocal_ = 0 if G_i_ = 0


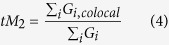


where tM_2_ = fraction of green-emitting pixels that also emit red, G_i,colocal_ = G_i_ if R_i_ > 0 and G_i,colocal_ = 0 if R_i_ = 0. MCC values range from 0 for no co-localization, to 1 for perfect co-localization^60^.

Autofluorescence due to excitation at the wavelengths used in this study was negligible (Supplementary Fig. S2).

According to the Stokes-Einstein equation, for a spherical molecule, diffusion coefficient is inversely proportional to the cube root of the molecular weight. This relationship was used to estimate theoretical value of the diffusion coefficient for some molecules.

### Materials

φ450-cAMP, φ575-cAMP, φ575-OMe-cAMP and φ450-free dye were either purchased or obtained as a gift from Biolog Life Science Institute (Bremen, Germany); MitoTracker Red was purchased from Life Technologies (Carlsbad, CA); EGFP from Biovision Inc. (Milpitas, CA), ST-Ht31 and St-Ht31P from Promega (Madison, WI). All other chemicals were purchased from Sigma-Aldrich (St. Louis, MO).

### Statistics

All data are expressed as the mean ± s.e.m. of the indicated number of independent experiments. Statistical significance (P < 0.05) was determined by Student’s t test.

## Additional Information

**How to cite this article**: Agarwal, S. R. *et al.* Mechanisms Restricting Diffusion of Intracellular cAMP. *Sci. Rep.* **6**, 19577; doi: 10.1038/srep19577 (2016).

## Supplementary Material

Supplementary Information
