# Supplementary material for: Mechanisms Restricting Diffusion of Intracellular cAMP
Source: Sci Rep. 2016 Jan 22;6:19577. doi: 10.1038/srep19577 (PMC4726171; doi:10.1038/srep19577)
Supplement: Supplementary Information [file srep19577-s1.pdf]

Mechanisms Restricting Diffusion of Intracellular cAMP

Shailesh R. Agarwal[1,*], Colleen E. Clancy[2], and Robert D. Harvey[1]

[1]Department of Pharmacology
University of Nevada School of Medicine
Reno, NV 89557

[2]Department of Pharmacology
University of California, Davis
Davis, CA 95616

*To whom correspondence should be addressed. Email: sagarwal@unr.edu

SUPPLEMENTARY INFORMATION

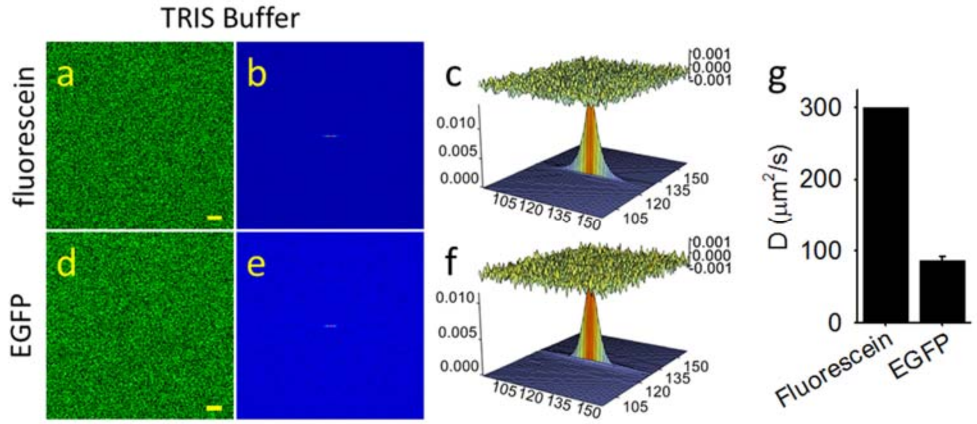

Supplementary Fig. S1. Calibration of microscope using RICS analysis of fluorescein and EGFP in Tris buffer.

Representative confocal images of fluorescein (a) and EGFP (d) in Tris buffer (scale bar 1 µm). (b, e) Average of the spatial correlation calculated for each of 100 images of fluorescein (b) and EGFP (e) in Tris buffer. Fit of the correlation functions for fluorescein (c) and EGFP (f). The plot at the top of those panels represents the difference between the autocorrelation and the fit. (g) Bar plots of the diffusion coefficient values for fluorescein and EGFP (average ± s.e.m.). The known value of D for fluorescein in Tris buffer (300 µm^2/sec) was fixed to obtain beam waist (ω_0). The diffusion coefficient for EGFP in Tris buffer was calculated using the ω_0 value obtained using fluorescein (n = 9).

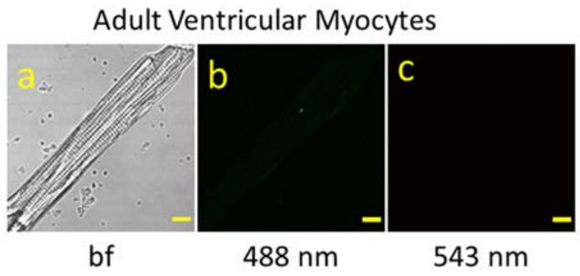

Supplementary Fig. S2. Control cardiac myocytes do not exhibit autofluorescence.

Representative bright field (a) and confocal images of control myocytes incubated with extracellular solution at 488 nm (b) and 543 nm (c). These experiments were repeated on three different days.

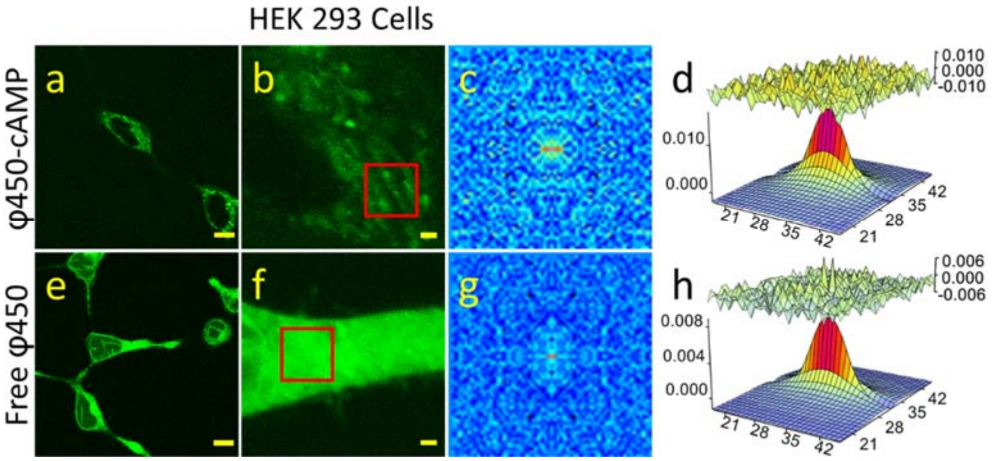

Supplementary Fig.S3. RICS analysis of φ450-cAMP and free φ450 in HEK293 cells. Representative confocal images of φ450-cAMP (a, b) and free φ450 (e, f) in HEK293 cells at low (a and e,scale bar 10 μm) and high (b and f, scale bar 1 μm) magnification. (c, g) Average of the spatial correlation calculated for a region of 64 X 64 pixels (indicated by the red box in b, f) for each of 100 images of φ450-cAMP (c) and free φ450 (g) in HEK293 cells. (d, h) Fit of the correlation functions. The plot at the top of those panels represents the difference between the autocorrelation and the fit.

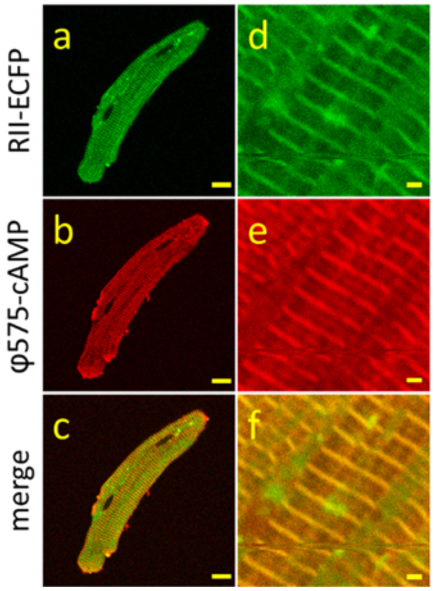

Supplementary Fig. S4. Co-localization of φ575-cAMP with the type II regulatory subunit (RII) of PKA in adult ventricular cardiac myocytes.

Confocal images of myocytes expressing RII-ECFP (a, d) or loaded with φ575-cAMP (b, e) at low (left panels, scale bar 10 μm) and high (right panels, scale bar 1 μm) magnification. The merged images (c, f) demonstrate a high degree of co-localization between RII-ECFP and φ575-cAMP. This example illustrates cells exhibiting a horizontal RII-EGFP expression pattern, in addition to the more commonly observed longitudinal pattern (see figure 4). Note that φ575-cAMP co-localizes with both. This pattern was observed in 3 out of 12 myocytes isolated from three different animals.

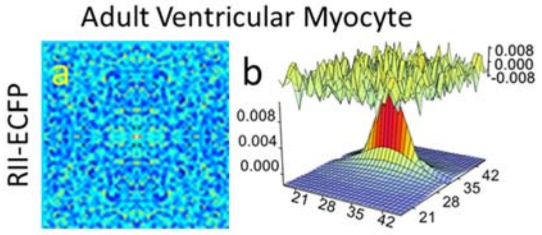

Supplementary Fig. S5. RICS analysis of RII-ECFP in an adult ventricular cardiac myocyte.

(a) Average of the spatial correlation calculated for a region of 64 X 64 pixels for each of 100 images of the RII-ECFP expressing myocyte. (b) Fit of the correlation functions for RII-ECFP. The plot at the top of that panel represents the difference between the autocorrelation and the fit.

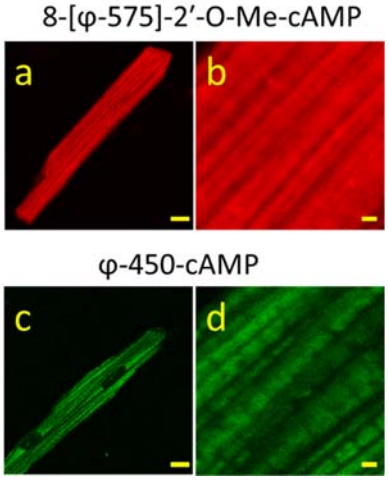

Supplementary Fig. S6. Confocal imaging of adult ventricular myocyte loaded with 8-[φ-575]-2′-O-Me-cAMP or φ450-cAMP.

Representative confocal images of a cardiac myocyte loaded with 8-[φ-575]-2′-O-Me-cAMP (upper panels, n = 36) or φ450-cAMP (lower panels) at low (a, c, scale bar 10 μm) and high (b, d, scale bar 1 μm) magnification. These experiments were repeated using myocytes isolated from three different animals.

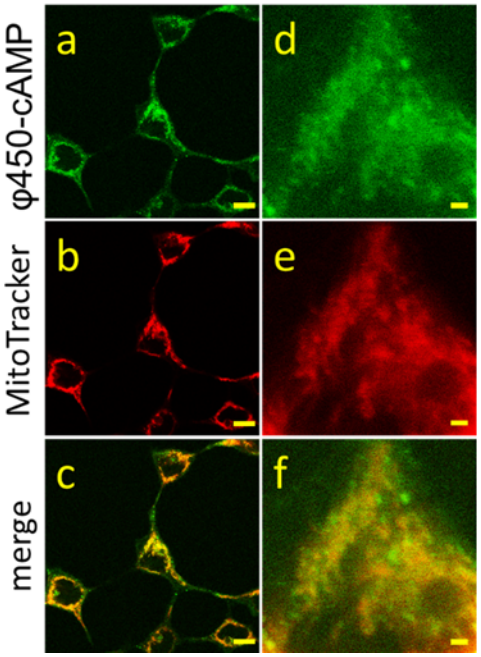

Supplementary Fig. S7. Co-localization of φ450-cAMP with MitoTracker red in HEK 293 cells. Representative confocal images of HEK 293 cells loaded with φ450-cAMP (a, d) and MitoTracker red (b, e) at low (a – c, scale bar 10 μm) and high (d – f, scale bar 1 μm) magnification. The merged images (c, f) demonstrate a high degree of co-localization of φ450-cAMP with MitoTracker red. PCC 0.42 ± 0.0042, tM1 0.58 ± 0.0076, tM2 0.60 ± 0.0064, n=13.

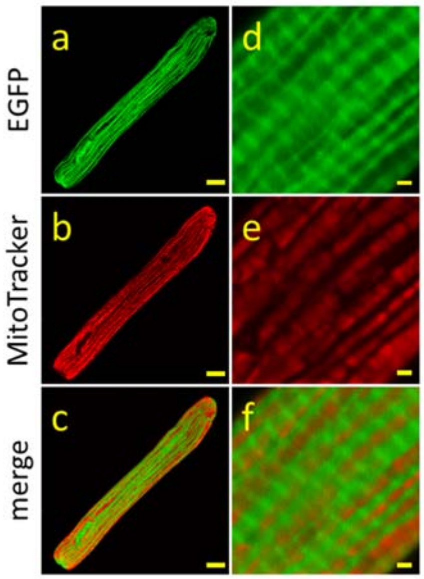

Supplementary Fig. S8. Confocal imaging of an EGFP-expressing adult ventricular cardiac myocyte loaded with MitoTracker red.

Representative confocal images of a cardiac myocyte expressing EGFP (a, d) and loaded with MitoTracker red (b, e) at low (a – c, scale bar 10 μm) and high (d – f, scale bar 1 μm) magnification. The merged images (c, f) of MitoTracker red with EGFP demonstrate that the EGFP expression is excluded from the regions that are occupied by the mitochondria in myocytes. PCC -0.38 ± 0.012, n=9.

Supplementary Table S1. Intracellular concentrations of various fluorophores obtained from RICS analysis in adult cardiac myocytes and HEK293 cells

Fluorophore	Myocytes		HEK293 cells	
	Range (nM)	Average (nM)	Range (nM)	Average (nM)
**[φ450-cAMP]**	180 – 960	639 ± 19	30 – 350	130 ± 6.1
**[EGFP]**	130 – 910	396 ± 64	40 – 690	245 ± 53
**[Free φ450]**	160 – 1010	676 ± 47	190 – 920	471 ± 53
**[Fluorescein]**	360 – 1050	749 ± 84	730 – 870	820 ± 44
